# *Lactobacillus delbrueckii* subsp. *lactis* CKDB001 Ameliorates Scopolamine-Induced Cognitive Impairment Through Metabolic Modulation

**DOI:** 10.3390/ijms262411804

**Published:** 2025-12-06

**Authors:** Hyerim Kim, Hyun Kim, Yeonmi Lee, Changho Park, Beomki Cho, Suyoung Son, Hyeyoung Kim, Gihyeon Kim, Jaeseong Park, Hansoo Park

**Affiliations:** 1 Genome and Company, Suwon 16229, Republic of Korea; hyerim.kim@genomecom.co.kr (H.K.); yeonmi@genomecom.co.kr (Y.L.); changho@genomecom.co.kr (C.P.); suyoung@genomecom.co.kr (S.S.); hyeyoung@genomecom.co.kr (H.K.); gihyeon@genomecom.co.kr (G.K.); 2 Department of Biomedical Science and Engineering, Gwangju Institute of Science and Technology (GIST), Gwangju 61005, Republic of Korea; hkim0719@gm.gist.ac.kr (H.K.); chobk1015@gm.gist.ac.kr (B.C.); pjs63788@gm.gist.ac.kr (J.P.)

**Keywords:** microbiome, *Lactobacillus*, Alzheimer’s disease, scopolamine, metabolomics

## Abstract

Microbiome-derived metabolites have emerged as key mediators of the gut–brain axis, influencing cognitive function and neuroprotection. This study investigated whether *Lactobacillus delbrueckii* subsp. *lactis* CKDB001 alleviates scopolamine-induced memory impairment through metabolic modulation, and how its effects compare with those of donepezil. ICR mice were administered CKDB001 or donepezil for 4–5 weeks and evaluated through behavioral, microbiome, metabolomic, and molecular analyses. CKDB001 significantly improved spatial working memory in a dose-dependent manner, with the high-dose group showing improvements comparable to those of the donepezil-treated group, while passive avoidance showed a non-significant but positive trend. Both CKDB001 and donepezil modulated gut microbial composition, leading to a partial divergence from the scopolamine-disrupted community structure, with CKDB001 inducing dose-dependent intestinal colonization. Metabolomic profiling revealed that both treatments increased tryptophan-derived indole metabolites and altered lipid and short-chain fatty acid metabolite profiles, although these effects were more pronounced in CKDB001-treated mice. At the molecular level, both CKDB001 and donepezil reduced hippocampal tau phosphorylation, downregulated glycogen synthase kinase-3 (GSK-3) signaling, enhanced intestinal tight-junction proteins, and partially normalized acetylcholinesterase activity, with CKDB001 restoring AChE levels more closely toward the normal control. Together, these findings suggest that CKDB001 mitigates cognitive deficits through coordinated modulation of microbial, metabolic, and neuronal pathways, offering a microbiome-based therapeutic approach that may provide benefits comparable to donepezil with potentially fewer limitations.

## 1. Introduction

Cognitive impairment, marked by memory loss and synaptic dysfunction, is a key feature of neurodegenerative diseases such as Alzheimer’s disease [1]. A well-established model for studying such deficits involves scopolamine, a muscarinic acetylcholine receptor antagonist that transiently disrupts cholinergic signaling and impairs memory [2]. This model closely mimics cholinergic dysfunction observed in dementia and is commonly used to evaluate cognitive-enhancing agents like acetylcholinesterase (AChE) inhibitors [3]. While AChE inhibitors such as donepezil can increase synaptic acetylcholine levels, their effects are temporary and often accompanied by side effects [4]. These limitations have driven interest in alternative approaches that target upstream regulators of cognition, including through modulation of the gut–brain axis.

Recent research has emphasized the influential role of the gut microbiota in regulating central nervous system (CNS) function through immune, endocrine, and metabolic pathways. The microbiota–gut–brain axis has emerged as a key area of study, with findings suggesting that imbalances in microbial communities can affect neuroinflammation, neurotransmitter synthesis, and the integrity of the blood–brain barrier [5,6,7]. Among microbial metabolites, tryptophan-derived compounds such as indole-3-lactic acid (ILA), indole-3-acetic acid (IAA), and indole-3-propionic acid (IPA) have shown potential in promoting neuroprotection by modulating host signaling and reducing inflammatory responses [8,9,10].

Probiotic strains from the genera *Lactobacillus* and *Bifidobacterium* have been reported to restore microbial balance, enhance intestinal barrier integrity, and improve cognitive performance in both aging and chemically induced impairment models [11,12]. Among these, strains such as *Lactobacillus helveticus* and *Lactobacillus johnsonii* have been shown to alleviate scopolamine-induced memory deficits by attenuating neuroinflammation [13,14]. Building on this concept, we previously demonstrated that *Lactobacillus delbrueckii* subsp. *lactis* CKDB001 exerts neuroprotective effects through aryl hydrocarbon receptor (AhR)-mediated pathways [10]. However, the metabolic mechanisms through which probiotics influence the gut–brain axis remain insufficiently elucidated. In particular, how tryptophan-derived metabolites affect tau phosphorylation and GSK-3 signaling is not yet fully understood, and whether CKDB001 can modulate cholinergic dysfunction and reshape microbiota–metabolite interactions in a scopolamine-induced model of cognitive impairment remains unclear.

In this study, we investigated whether oral administration of CKDB001 could alleviate scopolamine-induced memory impairment via gut–brain axis regulation. By integrating behavioral tests, microbiome analysis, metabolomics, and molecular profiling, we aimed to elucidate microbiota-mediated metabolic mechanisms underlying cognitive restoration.

## 2. Results

### 2.1. CKDB001 Improves Cognitive Function

**Figure 1:**
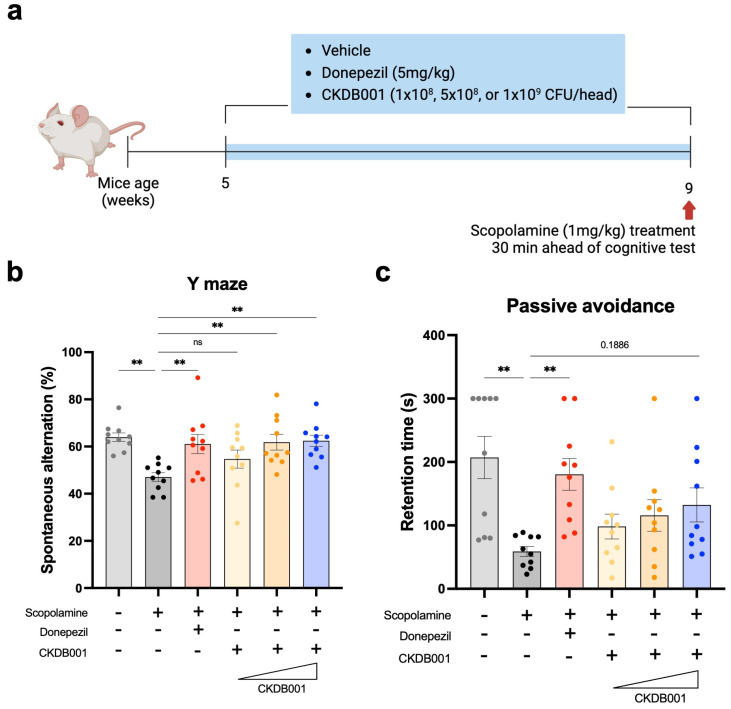
*Lactobacillus delbrueckii* subsp. *lactis* CKDB001 ameliorates scopolamine-induced cognitive impairment in mice. (**a**) Schematic timeline of the experimental design. ICR mice (5 weeks old) received vehicle, donepezil (5 mg/kg/day), or CKDB001 (1 × 10^8^, 5 × 10^8^, or 1 × 10^9^ CFU/day) orally for 4 weeks. Scopolamine (1 mg/kg, i.p.) was administered 30 min before behavioral tests (n = 10 per group). (**b**) Percentage of spontaneous alternation in the Y-maze test evaluating spatial working memory. (**c**) Latency to enter the dark compartment in the passive avoidance test assessing memory retention. Data are expressed as the mean ± SEM. Statistical significance was determined by One-way ANOVA with Dunnett’s test for multiple comparisons was used for statistical analysis (**b**). The Kruskal–Wallis test with Dunn’s test for multiple comparisons was used for (**c**). (** *p* < 0.01).

To evaluate the cognitive-enhancing effects of *Lactobacillus delbrueckii* subsp. *lactis* CKDB001, we employed a scopolamine-induced memory impairment mouse model. After a one-week acclimation period, mice were orally administered CKDB001 at doses of 1 × 10^8^, 5 × 10^8^, or 1 × 10^9^ CFU/mouse/day, or donepezil (5 mg/kg/day) for four weeks. Scopolamine (1 mg/kg) was intraperitoneally injected 30 min before behavioral testing, and all mice underwent Y-maze and passive avoidance tests to assess short-term spatial working memory and memory retention, respectively (Figure 1a). In the Y-maze test, scopolamine treatment markedly reduced the spontaneous alternation percentage compared with the normal control group, confirming impaired working memory. Oral administration of CKDB001 significantly restored alternation performance in a dose-dependent manner (Figure 1b). Both the 5 × 10^8^ and 1 × 10^9^ CFU/day CKDB001 groups exhibited improvements comparable to those of the donepezil-treated group, whereas the 1 × 10^8^ CFU/day group showed a modest but non-significant trend toward recovery. In the passive avoidance test, the latency to enter the dark chamber showed a dose-dependent increase in CKDB001-treated groups compared to the scopolamine-only group, while the differences were not statistically significant (*p* = 0.1886, Figure 1c).

Taken together, these findings demonstrate that CKDB001 ameliorates scopolamine-induced memory deficits in a dose-dependent manner, suggesting that higher doses of CKDB001 (≥5 × 10^8^ CFU/day) may effectively restore cognitive performance.

### 2.2. CKDB001 Alters Gut Microbial Composition and Establishes Colonization

**Figure 2:**
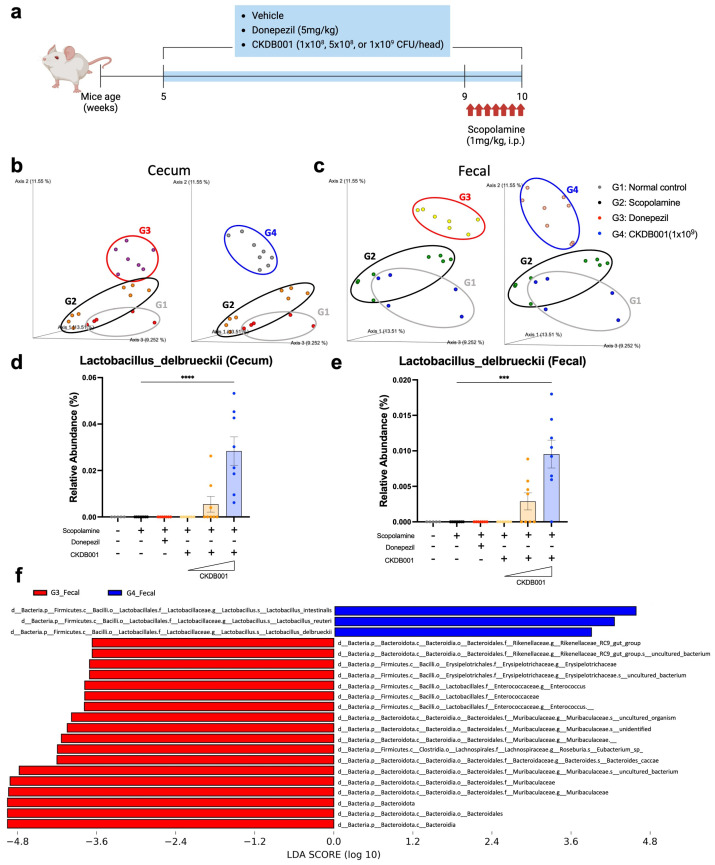
CKDB001 modulates gut microbiota composition and establishes intestinal colonization. (**a**) Experimental design for microbiome and molecular analyses following CKDB001 or donepezil administration and scopolamine injection. (**b**,**c**) Principal coordinate analysis (PCoA) of β-diversity based on 16S rRNA sequencing of cecal (**b**) and fecal (**c**) microbiota showing distinct clustering among groups (n = 5–8 per group). Distinct clustering was observed among normal (G1), scopolamine (G2), donepezil (G3), and CKDB001-treated(1 × 10^9^ CFU/day) groups (G4). (**d**,**e**) Relative abundance of *Lactobacillus delbrueckii* in cecal (**d**) and fecal (**e**) samples indicating dose-dependent colonization of CKDB001 (n = 5–8 per group). (**f**) Linear discriminant analysis effect size (LEfSe) comparing the fecal microbiota between the donepezil-treated (G3) and CKDB001-treated (G4) groups. Data are presented as the mean ± SEM. Statistical analysis was performed using PERMANOVA for beta-diversity and Kruskal–Wallis followed by Dunn’s test for relative abundance (*** *p* < 0.001, **** *p* < 0.0001).

To investigate the microbiome-associated effects of CKDB001, mice were first acclimated and then administered oral treatments for five weeks with graded doses of *Lactobacillus delbrueckii* subsp. *lactis* CKDB001 (1 × 10^8^, 5 × 10^8^, or 1 × 10^9^ CFU/day) or donepezil (5 mg/kg/day). During the final week, all mice received daily intraperitoneal injections of scopolamine to induce memory impairment and were sacrificed 30 min after the final injection. Cecal and fecal samples were collected for subsequent biomarker and molecular analyses (Figure 2a). Among these analyses, 16S rRNA gene sequencing was performed using cecal and fecal samples to characterize the effects of CKDB001 on gut microbial composition. Beta-diversity analysis revealed distinct clustering among the normal (G1), scopolamine-treated (G2), and donepezil-treated (G3) groups, indicating that both scopolamine and donepezil altered the overall gut microbial structure (Figure 2b,c). In CKDB001-treated mice, the microbial community exhibited a gradual divergence from the scopolamine-treated (G2) group in a dose-dependent manner, forming a distinct cluster at the highest dose (1 × 10^9^ CFU/day; G4) (Figure 2b,c and Supplementary Figure S1a,b). This separation pattern suggests that CKDB001 induced a unique reorganization of the gut microbial composition rather than recapitulating the donepezil-associated profile. Taxonomic profiling of both cecal and fecal samples further demonstrated that *Lactobacillus delbrueckii* was not detected in the normal, scopolamine, or donepezil groups but became detectable at the 5 × 10^8^ CFU/day dose and reached its highest relative abundance in the 1 × 10^9^ CFU/day group (Figure 2d,e). Comparative analysis of fecal microbiota between the donepezil-treated (G3) and high-dose CKDB001 (G4) groups revealed a marked enrichment of *Lactobacillus reuteri*, *Lactobacillus delbrueckii*, and *Lactobacillus intestinalis* in the G4 group (Figure 2f). Similarly, comparison between the scopolamine-treated (G2) and CKDB001-treated (G4) groups showed that *L. delbrueckii* and *L. intestinalis* were substantially increased following CKDB001 administration (Supplementary Figure S1c). These results confirm successful intestinal colonization of CKDB001 and indicate that high-dose treatment not only facilitated engraftment the administered *L. delbrueckii* strain but also promoted the proliferation of other beneficial *Lactobacillus* species, thereby contributing to broader remodeling of the gut microbial community.

### 2.3. CKDB001 Modulates Tryptophan-Derived Metabolites and Lipid Remodeling

**Figure 3:**
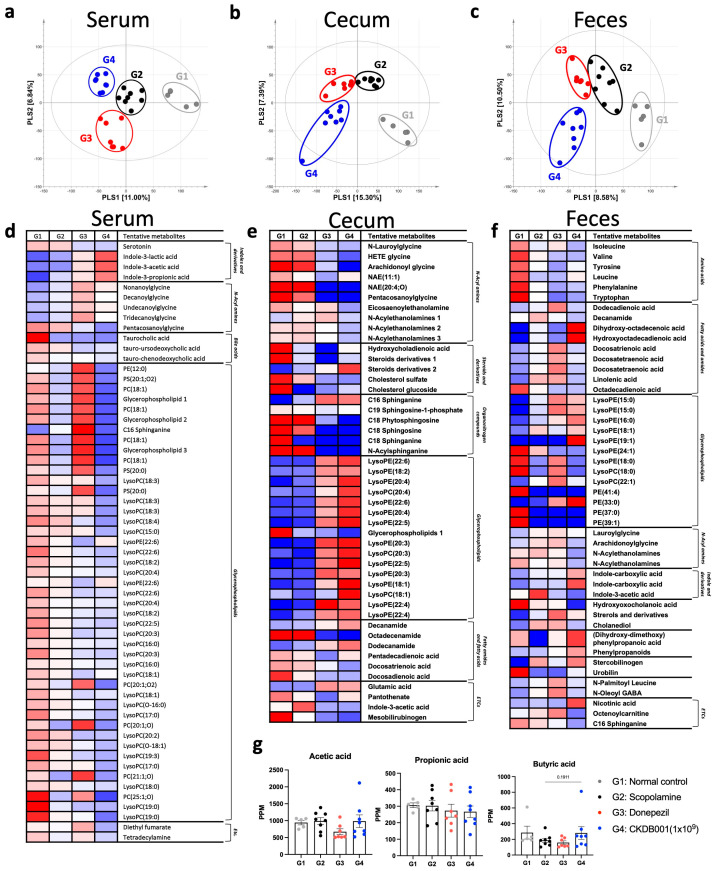
CKDB001 reshapes serum, cecal, and fecal metabolic profiles in scopolamine-treated mice. (**a**–**c**) Partial least squares-discriminant analysis (PLS-DA) score plots of serum (**a**), cecal (**b**), and fecal (**c**) metabolites demonstrating distinct metabolic clustering among groups. Distinct clustering was observed among normal (G1), scopolamine (G2), donepezil (G3), and CKDB001-treated (1 × 10^9^ CFU/day) groups (G4). (**d**–**f**) Heatmaps of significantly altered metabolites in serum (**d**), cecum (**e**), and feces (**f**). (**g**) Concentrations of short-chain fatty acids (acetic acid, propionic acid, and butyric acid) in fecal samples. Data are presented as mean ± SEM. Differences among four groups (G1–G4) were analyzed using the nonparametric Kruskal-Wallis test, and the difference between G2 and G4 was further evaluated using the Mann-Whitney U test. Metabolites with VIP > 1.0 in PLS-DA model were identified as major contributors distinguishing the group (n = 5–8 per group).

To elucidate the metabolic mechanisms associated with CKDB001 treatment, untargeted metabolomic profiling was performed using serum, cecal, and fecal samples. Partial least squares-discriminant analysis (PLS-DA) revealed clear separation among the normal (G1), scopolamine-treated (G2), donepezil-treated (G3), and CKDB001-treated (G4) groups across all sample types, indicating distinct metabolic reprogramming induced by CKDB001 (Figure 3a–c). In serum, CKDB001 administration markedly increased the levels of tryptophan-derived indole metabolites, including indole-3-lactic acid (ILA), indole-3-acetic acid (IAA), and indole-3-propionic acid (IPA), compared with the scopolamine group (Figure 3d). Donepezil treatment also increased these indole metabolites, although to a lesser extent than CKDB001. These metabolites are known to be produced by beneficial gut bacteria such as *Lactobacillus* and *Bifidobacterium* and have been reported to exert neuroprotective effects by activating the aryl hydrocarbon receptor (AhR) pathway, suppressing neuroinflammation, and enhancing brain-derived neurotrophic factor (BDNF) expression [10,15,16]. In the cecum and feces, several lipid metabolites, including lysophosphatidylcholines (LysoPCs) and lysophosphatidylethanolamines (LysoPEs), were differentially regulated by CKDB001 treatment (Figure 3e,f). These lipid alterations suggest remodeling of membrane-associated phospholipid metabolism, which may contribute to epithelial integrity and maintenance of gut barrier function [17,18,19,20,21,22]. Analysis of short-chain fatty acids (SCFAs) revealed no significant differences in acetic acid or propionic acid levels among groups. However, butyric acid levels exhibited a decreasing trend in the scopolamine (G2) and donepezil (G3) groups and were restored to near-normal levels in CKDB001-treated mice (Figure 3g). Although this change did not reach statistical significance, the upward trend of butyrate implies partial restoration of gut microbial metabolic activity associated with neuroprotective and anti-inflammatory signaling [23,24], potentially underlying the cognitive improvements observed in CKDB001-treated mice.

Collectively, these findings indicate that CKDB001 supplementation promotes the production of indole metabolites and modulates lipid- and SCFA-associated metabolic pathways, suggesting a potential metabolic link between CKDB001-induced microbial remodeling and the cognitive improvements observed in behavioral tests.

### 2.4. Formatting of Mathematical Components

**Figure 4:**
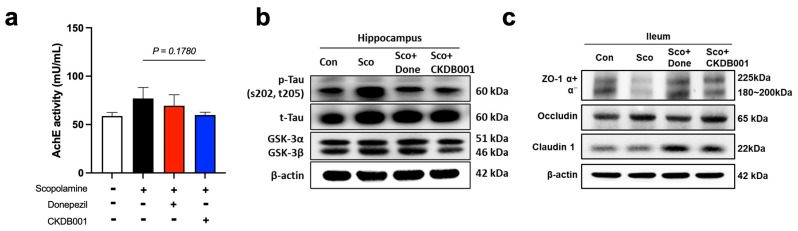
CKDB001 reduces hippocampal tau phosphorylation and restores gut barrier integrity impaired by scopolamine. (**a**) Acetylcholinesterase (AChE) activity in brain tissue indicating partial recovery of cholinergic function following CKDB001 treatment (n = 5–8 per group). Data are expressed as the mean ± SEM. Statistical number was determined by unpaired t-test with Welch’s correction. (**b**) Western blot analysis of hippocampal p-Tau (Ser202/Thr205), total Tau, GSK-3α, and GSK-3β expression levels. The band were shown by pooling samples (n = 5–8 per group) at equal volume for each group. (**c**) Western blot analysis of ileal tight junction proteins ZO-1 (α^+^/α^−^ isoforms), Occludin, and Claudin-1, demonstrating improvement in intestinal barrier integrity. The band were shown by pooling samples (n = 5–8 per group) at equal volume for each group.

Given that indole-derived metabolites produced by *Lactobacillus* species can attenuate inflammation and improve neuronal function [10,15,25], we next investigated whether CKDB001 influences cholinergic activity and gut–brain axis–related molecular pathways. Analysis of acetylcholinesterase (AChE) activity revealed that CKDB001 administration decreased AChE levels compared with the scopolamine-treated group, although the difference did not reach statistical significance (*p* = 0.178; Figure 4a). Nevertheless, the AChE activity in the CKDB001 group approached that of the normal group, suggesting a partial restoration of cholinergic signaling. This trend implies that CKDB001 may confer cognitive benefits indirectly through multiple mechanisms, including anti-inflammatory and metabolic regulation, rather than direct AChE inhibition, as observed with donepezil. In the hippocampus, CKDB001 or donepezil treatments markedly reduced phosphorylated tau (p-Tau [Ser202/Thr205]) levels while increasing total tau (t-Tau) expression. Concurrently, GSK-3β protein level was reduced (Figure 4b and Supplementary Figure S2a), indicating suppression of the GSK-3-mediated tau-hyperphosphorylation pathway. In the ileum, the expression of tight-junction proteins ZO-1 (α^+^/α^−^ isoforms), Occludin, and Claudin-1 was notably upregulated following CKDB001 or donepezil treatments (Figure 4c and Supplementary Figure S2b), suggesting restoration of epithelial barrier integrity disrupted by scopolamine and attenuation of systemic inflammation.

Taken together, these data suggest that CKDB001 confers neuroprotective benefits through the gut–brain axis by reinforcing intestinal barrier function, mitigating inflammatory and tau-related pathology, and partially restoring cholinergic signaling. This integrated mechanism aligns with the preceding findings and highlights CKDB001 as a promising microbiome-based therapeutic candidate for memory impairment that exerts effects comparable to donepezil through broader microbiota- and metabolite-driven pathways.

## 3. Discussion

The present study demonstrates that *Lactobacillus delbrueckii* subsp. *lactis* CKDB001 ameliorates scopolamine-induced cognitive impairment through coordinated modulation of the gut microbiota, microbial metabolites, lipid remodeling, intestinal barrier integrity, and both cholinergic and tau-associated signaling pathways. In contrast to donepezil, which primarily exerts its effects via direct inhibition of acetylcholinesterase (AChE), CKDB001 appears to act at an upstream level by modulating gut-derived metabolic and inflammatory pathways that, in turn, influence neuronal function.

CKDB001 supplementation markedly reshaped the gut microbial community structure (Figure 2 and Supplementary Figure S1). Notably, comparative analysis between the donepezil (G3) and CKDB001 (G4) groups revealed a higher relative abundance of *Lactobacillus delbrueckii*, *Lactobacillus reuteri*, and *Lactobacillus intestinalis* in the CKDB001 group (Figure 2f). *L. reuteri* is known to produce indole metabolites that activate the aryl hydrocarbon receptor (AhR), suppress intestinal [26] and neuroinflammation, and improve cognitive performance [27]. Similarly, *L. delbrueckii* has been reported to attenuate intestinal inflammation [28] and enhance cognitive function [29], underscoring its potential role as a neuroprotective and gut-modulating probiotic strain. Consistent with these observations, our previous study demonstrated that *L. reuteri* and CKDB001 ameliorate neuroinflammation and preserve cognitive function via indole–AhR signaling [10]. Furthermore, emerging evidence indicates that *L. intestinalis* enhances epithelial–phagocyte interactions to maintain mucosal integrity and restrict bacterial translocation [30], suggesting a possible contribution to gut homeostasis in the present model. In addition, the dose-dependent behavioral (Figure 1b,c) and microbiome changes (Figure 2b,c and Supplementary Figure S1a,b) observed in CKDB001-treated mice suggest that extending the treatment duration may further clarify the durability and long-term stability of CKDB001-induced microbial and metabolic remodeling. Future studies incorporating longer administration periods will help determine whether these effects persist or become further strengthened over time.

A defining metabolic signature of CKDB001 treatment was the elevation of tryptophan-derived indole metabolites, including indole-3-lactic acid (ILA), indole-3-acetic acid (IAA), and indole-3-propionic acid (IPA) (Figure 3d). Scopolamine treatment is known to induce oxidative stress, contributing to cognitive impairment [31]. However, microbiota-derived indole metabolites exert antioxidant effects through distinct mechanisms. IPA has been reported to alleviate oxidative stress in the brain by scavenging free radicals and enhancing antioxidant defense systems, including glutathione [32,33], whereas ILA has been shown to activate Nrf2-dependent pathways in intestinal tissue to reduce oxidative stress [34]. In addition, these metabolites are recognized AhR ligands that suppress NF-κB-mediated inflammatory signaling and reduce TNF-α and IL-6 production, thereby promoting a neuroprotective and anti-inflammatory environment [9,10,25]. Inflammatory states have been associated with increased AChE activity in the brain [35,36], whereas AhR activation has been reported to downregulate AChE expression in neuronal cell models [37]. Consistent with these findings, CKDB001 administration tended to normalize AChE activity in scopolamine-treated mice (Figure 4a). Collectively, these results suggest that CKDB001-induced increases in indole metabolites may activate AhR signaling, thereby attenuating inflammation and oxidative stress, and indirectly modulating AChE activity to support cholinergic function. Our prior work showing that CKDB001 elevates ILA levels, activates AhR, suppresses neuroinflammation, and preserves cognition further substantiates this mechanism [10].

Beyond tryptophan metabolism, CKDB001 also modulated lipid metabolic pathways, particularly by increasing lysophosphatidylcholine (LysoPC) and lysophosphatidylethanolamine (LysoPE) levels in the cecum and feces (Figure 3e,f). The intestinal mucus layer and epithelial membrane are enriched in phosphatidylcholine (PC) and LysoPC, which form a hydrophobic barrier that protects the epithelium from luminal toxins and microbial insults [17,18]. Clinical observational studies in patients with inactive ulcerative colitis have shown significantly reduced PC and LysoPC levels in rectal mucus, which correlate with impaired mucosal defense and persistent inflammation [17,18,19]. Similarly, reductions in LysoPE levels have been implicated in intestinal inflammation and epithelial injury [20,21], whereas oral LysoPE supplementation has been shown to alleviate peritonitis by decreasing pro-inflammatory mediators (IL-1β, IL-6, and TNF-α) while increasing the anti-inflammatory cytokine IL-10 [22]. In line with these findings, recent studies demonstrate that dietary lysophospholipid supplementation strengthens intestinal barrier integrity and upregulates tight-junction proteins such as ZO-1, supporting the role of lysophospholipids in epithelial repair [38]. Thus, the elevated LysoPC and LysoPE levels observed in CKDB001-treated mice likely reflect active phospholipid turnover associated with epithelial membrane remodeling and regeneration, rather than passive accumulation. Supporting this interpretation, LysoPC and LysoPE levels were reduced in serum (Figure 3d), but increased in the intestinal compartment (Figure 3e,f), suggesting preferential local utilization of lysophospholipids for epithelial repair and tight-junction restoration, rather than systemic release. In addition, elevated nicotinic acid levels observed in the fecal metabolome of CKDB001-treated mice may further support improved mucosal and immune function. Previous studies have reported that dietary nicotinic acid (niacin) strengthens epithelial integrity and suppresses intestinal inflammation [39,40], indicating that increased fecal niacin may contribute to enhanced intestinal immune homeostasis in the present model. Consistently, CKDB001 supplementation significantly upregulated intestinal tight-junction proteins (ZO-1, Occludin, and Claudin-1), indicating recovery of epithelial barrier integrity disrupted by scopolamine (Figure 4c). These findings collectively suggest that CKDB001 enhances gut barrier homeostasis, at least in part through lysophospholipid-mediated membrane remodeling, thereby contributing to its overall neuroprotective effect.

In addition to its effects on epithelial barrier restoration, CKDB001 further reduced hippocampal tau phosphorylation at Ser202/Thr205 and decreased GSK-3α/β expression (Figure 4b). Neuroinflammation is a key pathological feature of Alzheimer’s disease, and pro-inflammatory cytokines are known to promote hyperphosphorylation of tau, partly through upregulation of tau-kinases such as GSK-3β [41,42,43]. Thus, the reduction in GSK-3α/β levels and p-tau burden observed in CKDB001-treated mice may reflect a downstream consequence of decreased inflammatory tone driven by indole-AhR signaling and improved barrier function limiting systemic inflammatory inputs. In support of this interpretation, CKDB001-treated mice displayed increased levels of several anti-inflammatory metabolites, including serum indole derivatives and cecal and fecal LysoPC, LysoPE, and nicotinic acid, which are known to modulate inflammatory pathways and promote mucosal homeostasis. Although causal relationships remain to be fully established, these findings support the concept that microbial-metabolite-mediated suppression of inflammation can indirectly modulate GSK-3 activity and tau pathology, contributing to the overall neuroprotective effect of CKDB001.

Together, these results support a model in which CKDB001 alleviates cognitive impairment not by direct AChE inhibition, but by targeting upstream microbiota–metabolite–host signaling networks. Through the coordinated regulation of indole-AhR pathways, lipid homeostasis, and epithelial barrier function, CKDB001 exerts a multifaceted neuroprotective mechanism distinct from conventional cholinesterase inhibitors, highlighting microbial metabolic modulation as a promising complementary or alternative therapeutic strategy for cognitive dysfunction.

## 4. Materials and Methods

### 4.1. Mice

All animal experiments were carried out in accordance with protocols approved by the Institutional Animal Care and Use Committee of NDIC (IACUC No. P221053, approved on 31 October 2022) and Chaon (IACUC No. CE24780, approved on 23 December 2024). All animals used in this study were maintained and handled according to the approved policies of the study protocols. Wild type male ICR mice were provided by Orient Bio (Gapyeong, Gyeonggi, Republic of Korea). For behavior test, the mice were orally administrated with *Lactobacillus delbrueckii* subsp. *lactis* CKDB001 (1 × 10^8^, 5 × 10^8^, or 1 × 10^9^ CFU/mouse/day) and donepezil (5 mg/kg/day) for 4 weeks after a week-acclimation period. The mice were intraperitoneally injected with scopolamine (1 mg/kg) 30 min before behavior test and all mice were conducted Y maze test and passive avoidance test. For biomarker and molecular analysis, the mice were orally administered with *L. delbrueckii* subsp. *lactis* CKDB001 (1 × 10^8^, 5 × 10^8^, or 1 × 10^9^ CFU/mouse/day) and donepezil (5 mg/kg/day) for 5 weeks after a week-acclimation period. The mice were intraperitoneally injected with scopolamine (1 mg/kg/day) for last one week and then all mice were sacrificed 30min after last scopolamine injection. Tissue samples were stored at −80 °C or were fixed with 10% formaldehyde solution at RT until analysis. The probiotic strain used in this study, *Lactobacillus delbrueckii* subsp. *lactis* CKDB001, was kindly provided by Chong Kun Dang Bio Corporation (Seoul, Republic of Korea). CKDB001 has been reported in multiple preclinical and clinical studies demonstrating its safety and biological functions, including hepatic lipid regulation, modulation of gut microbial composition, and cognitive improvement [10,29,44,45,46]. These studies include evaluations in nonalcoholic fatty liver disease models [44], randomized clinical trials in mild cognitive impairment [29], and mechanistic assessments in the 5xFAD Alzheimer’s disease mouse model [10].

### 4.2. Y-Maze Test

The Y maze test was performed to evaluate short-term spatial working memory, in which higher memory performance is indicated when the subject avoids re-entering the same arm that was just explored. The Y-maze consisted of three arms, each measuring 40 cm in length, 3 cm in width, and 15 cm in height, with three arms positioned at 120° angles from each other. Each subject was allowed an 8-min exploration period, and the final outcome was expressed as the spontaneous alternation percentage (Spontaneous alternation, %), calculated using the following formula: spontaneous alternation (%) = [(The number of triplet)/(Total arm entries − 2)] × 100. Data were analyzed using EthoVision XT16 (Noldus, Leesburg, VA, USA).

### 4.3. Passive Avoidance Test

The passive avoidance test was performed to assess cognitive function based on the innate tendency of rodents to prefer dark environments. The apparatus consisted of two chambers of equal size: a light chamber and a dark chamber. The dark chamber was equipped with a foot-shock system. When the animal entered the dark chamber from the light chamber, a foot shock (0.5 mA, 3 s) was delivered. After 24 h, each animal was placed again in the light chamber, and the latency to enter the dark chamber was recorded as a measure of memory retention, with a maximum latency time of 300 s.

### 4.4. 16S rRNA Sequencing and Data Analysis

Raw paired-end sequencing reads were processed using QIIME2 version 2024.5. Briefly, quality filtering, denoising, and chimera removal were performed with the DADA2 algorithm implemented within the QIIME2 pipeline to generate amplicon sequence variants (ASVs). Phylogenetic relationships were constructed using the qiime phylogeny align-to-tree-mafft-fasttree command, which applies the MAFFT alignment and FastTree algorithms. A rarefaction depth of 70,577 reads per sample was selected based on the minimum sequencing depth required to retain all samples. Alpha and beta diversity metrics were calculated using the core-metrics-phylogenetic pipeline in the QIIME2 diversity plugin. Beta diversity was assessed based on Bray–Curtis dissimilarity and visualized using principal coordinate analysis (PCoA). Statistical significance of community dissimilarities among groups was evaluated using permutational multivariate analysis of variance (PERMANOVA) with 999 permutations, implemented in the beta-group-significance function of QIIME2. Taxonomic classification was performed using the SILVA 138 reference database. The V3–V4 hypervariable regions were extracted, and a naïve Bayes classifier implemented in QIIME2 was trained for taxonomy assignment. Taxonomic compositions were summarized at multiple taxonomic ranks, including the species level. To identify taxa that were differentially abundant among groups, linear discriminant analysis effect size (LEfSe) was applied (version 1.0). Features with a linear discriminant analysis (LDA) score > 2.0 were considered significantly enriched.

### 4.5. Metabolite Analysis

#### 4.5.1. Sample Preparation for Untargeted Metabolomics

Mouse serum (70 μL) was extracted with methanol including internal standard solution (2-chloro-L-phenylalanine, 1 mg/mL in water). After vortex-mixing for 1 min, ultrasonic extraction on ice was performed for 10 min (Power sonic 510, Hwasin Tech., Seoul, Republic of Korea) and the mixture was centrifuged at 4 °C, 13,000× *g* for 15 min (5424R, Eppendorf, Hamburg, Germany). The supernatant was filtered through a 0.2 μm polytetrafluoroethylene (PTFE) filter (Part no. 5191–5916, Agilent Technologies, Santa Clara, CA, USA) and dried in a vacuum concentrator (VS-802F, Visionbionex, Bucheon, Gyeonggi-do, Republic of Korea). Feces and cecum (100 mg, each) were extracted with methanol including the same internal standard solution. Samples were homogenized at 5 m/s for 2 min (Fastprep-24, MP Biomedicals, Irvine, CA, USA). Subsequent steps including vortex, sonication, and centrifuge were identical to serum. The supernatants were filtered and evaporated to dryness under vacuum. The dried extracts were reconstituted to appropriate volumes for metabolite analysis.

#### 4.5.2. Ultra High-Performance Liquid Chromatography-Tandem Mass Spectrometry (UHPLC-MS/MS) Analysis

Untargeted metabolomics was performed on a UHPLC system (Vanquish, Thermo Fisher Scientific, Waltham, MA, USA). Chromatographic separation was carried out on an Acquity UPLC BEH C18 column (100 mm × 2.1 mm, 1.7 μm particle size, Waters, Milford, MA, USA) maintained at 40 °C. Mobile phase A was water with 0.1% formic acid and mobile phase B was acetonitrile with 0.1% formic acid. The flow rate was 0.3 mL/min and the injection volume was 5 μL. The gradient program was set as follows: the composition was held at 5% B for 1.0 min, linearly increased to 100% for 1.0 to 10.0 min, held at 100% B from 10.0 to 11.0 min, returned to 5% B over 0.1 min, and re-equilibrated at 5% B until 13.0 min. The total run time was 13 min. For MS part, the UHPLC was coupled to Orbitrap Eclipse Tribrid mass spectrometer (Thermo Fisher Scientific, Waltham, MA, USA) equipped with a HESI-II source. MS data were acquired in positive and negative ion modes over *m*/*z* 70–1000, with full-scan spectra collected in the Orbitrap (resolution 60,000). Source settings were spray voltage +3.7 kV/−2.5 kV, vaporizer temperature 350 °C, and ion transfer tube temperature 300 °C.

#### 4.5.3. Data Analysis

UHPLC-MS/MS data were acquired with Xcalibur software (version 2.00, Thermo Fisher Scientific, Waltham, MA, USA). Raw files were converted to ABF (*.abf) format using ABFConverter (version 1.3.8802; https://www.reifycs.com/abfconverter/index; accessed on 2 December 2025). After conversion, data collection, retention time correction, peak detection, alignment, normalization, and accurate masses were determined using MS-dial software (version 5.1.230912, Riken, Kanagawa, Japan). The alignment data was exported to Excel files (Microsoft, Redmond, WA, USA). Multivariate statistical analyses were conducted using SIMCA-P+ software (version 18.0, Umetrics, Umea, Sweden). Principal component analysis (PCA) and partial least squares discrimination analysis (PLS-DA) were preformed to compare different metabolites among experimental groups. The significance of the PLS-DA model was defined by analysis of variance testing of cross-validated predictive residuals (CV-ANOVA) in the SIMCA-P+ program. Discriminative variables were selected based on variable importance in the projection (VIP) value of the PLS-DA. The selected metabolites obtained from UHPLC-MS/MS were tentatively identified based on various data comparing their retention time (min), mass spectrum (*m*/*z*), an MS fragment pattern with those for standard compounds analyzed under identical conditions, and various, available databases including the Human Metabolome Database (HMDB), PubChem, LIPIDMAPS, in house libraries. The level of identification was assigned according to the Metabolomics Standards Initiative (MSI) guidelines, with most annotations corresponding to tentative (Level 2) identifications. Statistical analyses were performed using GraphPad (version 10.6.0, Prism, La Jolla, CA, USA). Significantly difference in metabolites were assessed using the Kruskal–Wallis and Mann–Whitney U tests. A comprehensive list of all untargeted metabolites detected in serum, cecal, and fecal samples is provided in Supplementary Table S1.

### 4.6. ELISA

For acetylcholinesterase activity analysis, proteins from brain tissues were isolated in lysis buffer containing 50 mM Tris–HCl (pH 8.0), 1% Triton X-100, 1 mM EDTA (pH 8.0) in 1× PBS and were measured using Acetylcholinesterase Assay Kit (Abcam, Cambridge, UK, ab138871) according to manufacturer’s instructions.

### 4.7. Western Blot Analysis

Brain and ileum tissues were transferred into Lysing Matrix D tubes and mechanically homogenized using a MP FastPrep-24 homogenizer (MP Biomedicals, USA) according to the manufacturer’s instructions. Proteins were isolated by lysing cells in lysis buffer containing 50 mM Tris–HCl (pH 7.4), 1% Triton X-100, 50 mM NaCl, 1 mM DTT, and 1 mM EGTA in the presence of protease and phosphatase inhibitor cocktails (1 mM). The protein concentration was measured using a BCA protein assay kit (Thermo Fisher Scientific, Waltham, MA, USA). Protein samples (10–30 μg) were transferred onto polyvinylidene fluoride membranes (Biorad, Hercules, CA, USA) after being separated by SDS-PAGE. The membranes were blocked with 5% BSA-TBST at room temperature for 1 h and then incubated with various primary antibodies at 4 °C overnight, followed by HRP-conjugated secondary antibodies at room temperature for 1 h. Finally, the proteins were detected using ECL chemiluminescent HRP substrate (Thermo Fisher Scientific) using a Bio-Rad ChemiDoc imaging system. The primary antibodies used in this study are listed in Supplementary Table S2.

### 4.8. Statistical Analysis

Statistical calculations were performed using GraphPad (version 10.6.1, Prism, La Jolla, CA, USA). For parametric data, one-way ANOVA with Dunnett’s test for multiple comparisons was utilized for statistical analysis. For non-parametric data, the Kruskal–Wallis test with Dunn’s test for multiple comparisons was employed. Additionally, the unpaired *t*-test was used for comparisons between two groups in parametric data, while the Mann-Whitney test was used for non-parametric data. Statistical significance is indicated by asterisks (*); * *p* < 0.05, ** *p* < 0.01, *** *p* < 0.001, and **** *p* < 0.0001.

## 5. Conclusions

This study demonstrates that *Lactobacillus delbrueckii* subsp. *lactis* CKDB001 ameliorates scopolamine-induced cognitive impairment by modulating multiple components of the gut–brain axis. CKDB001 improved behavioral performance, reshaped gut microbiota composition, increased indole-derived metabolites and lysophospholipids, reinforced intestinal barrier integrity, reduced hippocampal tau phosphorylation and GSK-3 activity, and partially normalized AChE function. Unlike conventional cholinesterase inhibitors, CKDB001 exerts its effects through integrated microbial and metabolic pathways rather than direct enzymatic inhibition. These findings position CKDB001 as a promising microbiome-based therapeutic candidate for cognitive dysfunction. Further studies are warranted to confirm causality and evaluate its translational potential in chronic neurodegenerative diseases such as Alzheimer’s disease.

## Data Availability

The original contributions presented in this study are included in the article/supplementary material. Further inquiries can be directed to the corresponding authors.

## Supplementary Materials

The following supporting information can be downloaded at: https://www.mdpi.com/article/10.3390/ijms262411804/s1.

## Abbreviations

The following abbreviations are used in this manuscript:

AChE	Acetylcholinesterase
CNS	Central nervous system
ILA	Indole-3-lactic acid
IAA	Indole-3-acetic acid
IPA	Indole-3-propionic acid
AhR	Aryl hydrocarbon receptor
PLS-DA	Partial least squares-discriminant analysis
BDNF	Brain-derived neurotrophic factor
LysoPCs	Lysophosphatidylcholines
LysoPEs	Lysophosphatidylethanolamines
SCFAs	Short-chain fatty acids
p-Tau	Phosphorylated tau
t-Tau	Total tau
