# Supplementary material for: Lactobacillus delbrueckii subsp. lactis CKDB001 Ameliorates Scopolamine-Induced Cognitive Impairment Through Metabolic Modulation
Source: Int J Mol Sci. 2025 Dec 6;26(24):11804. doi: 10.3390/ijms262411804 (PMC12732984; doi:10.3390/ijms262411804)
Supplement: Supplementary file 1 [file ijms-26-11804-s001.zip › Supplementary Figures.docx]

**Supplementary Figures**

**Figure S1**


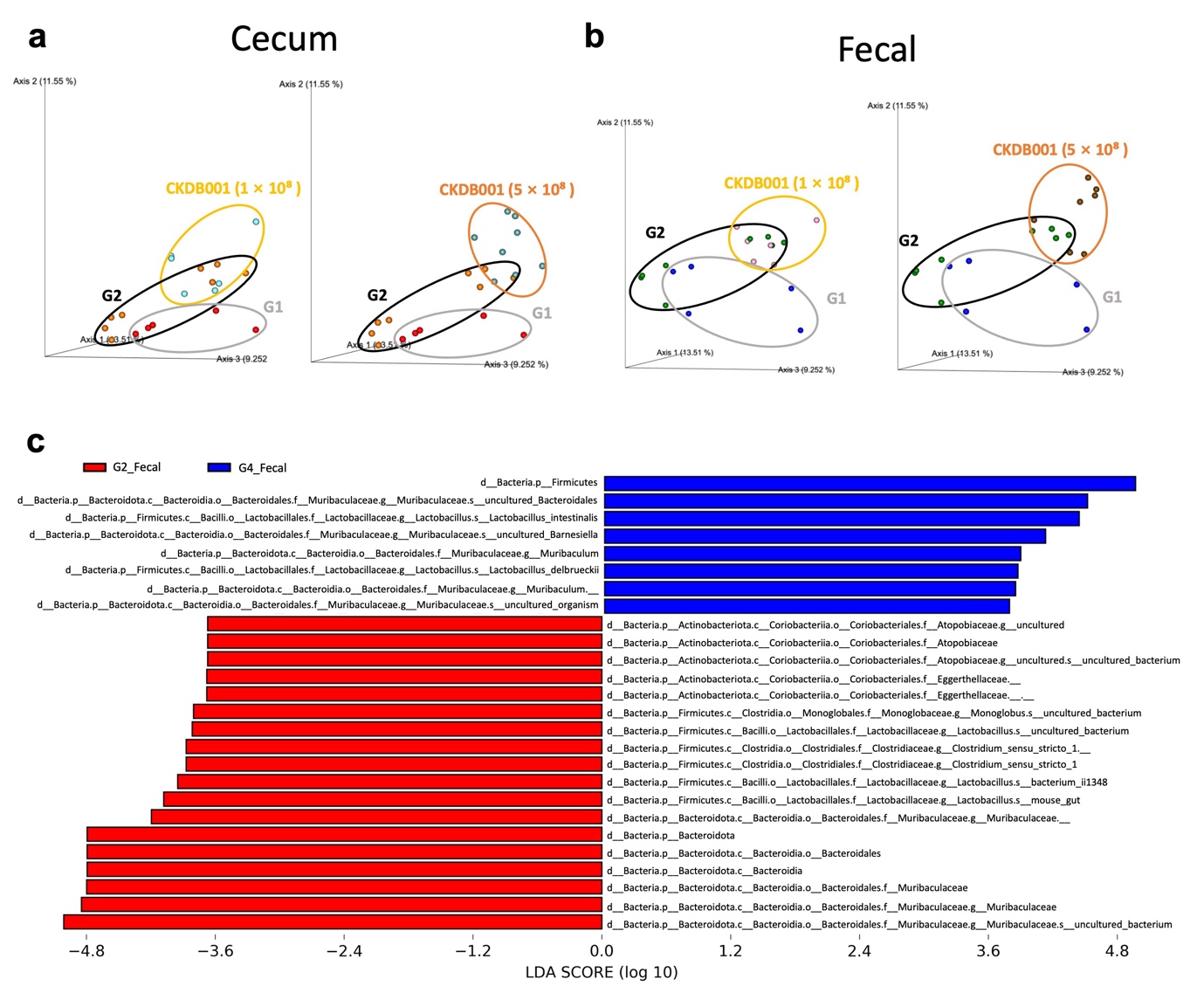


**Figure S1.** Alterations in gut microbial composition following CKDB001 treatment in scopolamine-induced mice. (a,b) Principal coordinate analysis (PCoA) of β-diversity based on 16S rRNA sequencing of cecal (a) and fecal (b) microbiota showing distinct clustering among groups (n = 5–8 per group). Distinct clustering was observed among normal (G1), scopolamine (G2), CKDB001-treated (1 × 10^8^and 5 × 10^8^ CFU/day) groups. (c) Linear discriminant analysis effect size (LEfSe) comparing the fecal microbiota between the scopolamine-treated (G2) and CKDB001-treated (G4) groups. Statistical analysis was performed using PERMANOVA for beta-diversity.

**Figure S2**


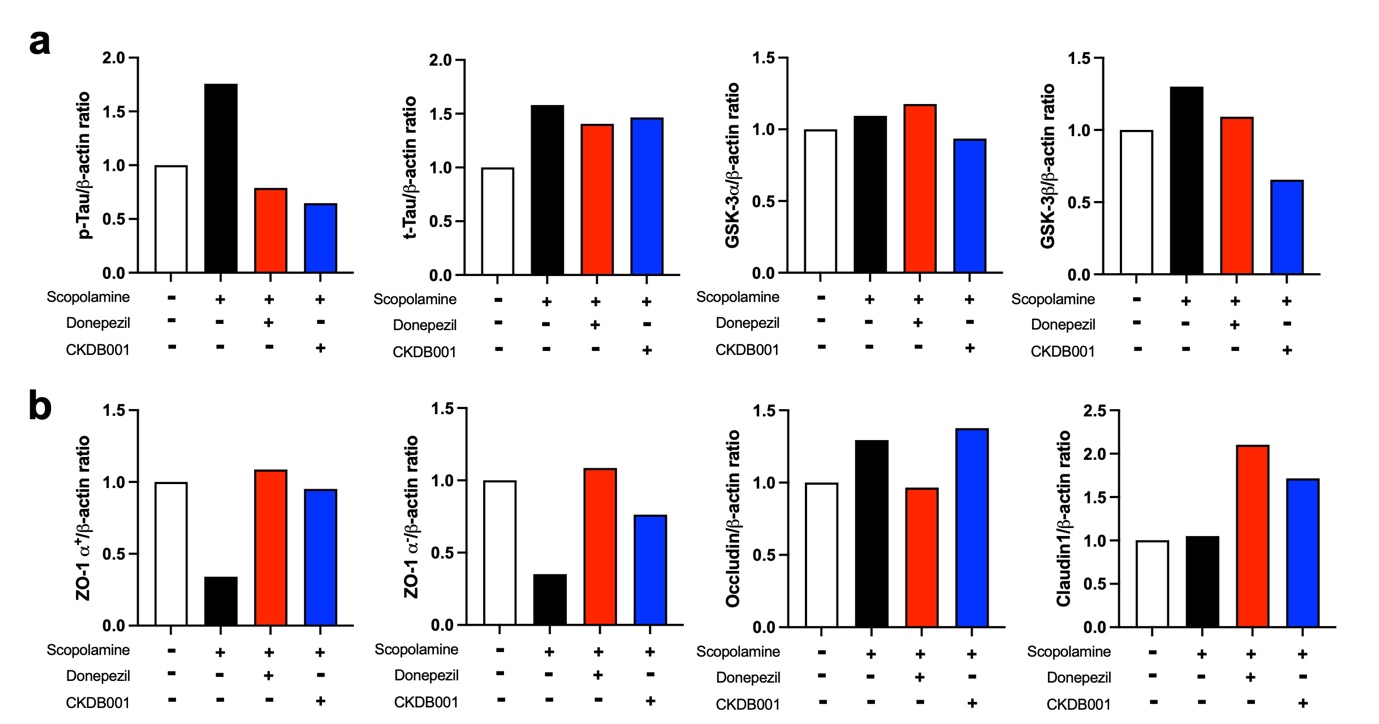


**Figure S2.** Quantitative analysis of western blot band intensity for hippocampal and ileal proteins. (a) Quantification of hippocampal proteins, including p-Tau (Ser202/Thr205), total Tau, GSK-3α, and GSK-3β. Band intensities were normalized to β-actin. (b) Quantification of ileal tight-junction proteins, including ZO-1 (α⁺/α⁻ isoforms), Occludin, and Claudin-1, corresponding to the western blot results shown in Figure 4c. Band intensities were normalized to β-actin. All quantification was performed using ImageJ software.
